# Emerging high-level ciprofloxacin-resistant *Salmonella enterica* serovar typhi haplotype H58 in travelers returning to the Republic of Korea from India

**DOI:** 10.1371/journal.pntd.0009170

**Published:** 2021-03-02

**Authors:** Eunkyung Shin, Jungsun Park, Hyun Ju Jeong, Ae Kyung Park, Kyoungin Na, Hyerim Lee, Jeong-hoon Chun, Kyu Jam Hwang, Chul-Joong Kim, Junyoung Kim

**Affiliations:** 1 Division of Bacterial Diseases, Korea Centers for Diseases Control and Prevention, Cheongju, Republic of Korea; 2 College of Veterinary Medicine, Chungnam National University, Daejeon, Republic of Korea; 3 Division of Infectious Diseases Control, Korea Centers for Diseases Control and Prevention, Cheongju, Republic of Korea; Pontificia Universidad Catolica de Chile, CHILE

## Abstract

In Korea, typhoid fever is a rare disease due to improved living standards. However, typhoid fever remains a major burden in developing countries and regions, such as India and Southeast Asia. In this study, we isolated *Salmonella* Typhi (*S*. Typhi) from eight patients with typhoid fever who were travelers returning from India. The strains isolated were characterized by antimicrobial susceptibility profiling and whole-genome sequencing (WGS) analysis. All strains were resistant to nalidixic acid and azithromycin. Among them, four isolates were highly resistant to ciprofloxacin (MIC ≥32 μg/ml); these strains have not been confirmed in Korea PulseNet DB. According to WGS, the ciprofloxacin-resistant strains belong to the global dominant multidrug-resistant (MDR) haplotype H58 (SNP *glpA* C1047T, *SptP* protein Q185* (premature stop codon)) and do not harbor the MDR plasmid. H58-associated SNPs in membrane and metabolism genes, including *yhdA*, *yajI*, *hyaE*, *tryE*, *rlpB* and *metH*, are present. Additionally, phylogenetic analysis assigned the H58 strains to sublineage II, whereas the non-H58 strains are closely related to haplotype H50. The presence of high-level ciprofloxacin-resistant *S*. Typhi haplotype H58 in Korea was first confirmed as due to influx from overseas via travelers. This study provides information about intercontinental drug-resistant transmission between countries and suggests that travelers need to be careful about personal hygiene.

## Author summary

Typhoid fever is a systemic human disease that involves gastroenteritis, fever, and severe diarrhea caused by *Salmonella enterica* serotype Typhi (*S*. Typhi) and requires prompt antibiotic treatment. Due to improved living standards, it has become a rare disease but is still prevalent in developing countries such as India and Southeast Asia. Most reported cases are related to travelers or immigrants from these regions. Because of treatment failure, serious morbidity, and economic loss, there is a global health issue with the emergence of antimicrobial-resistant strains associated with fluoroquinolones and third-generation cephalosporins. Of antimicrobial-resistant *S*. Typhi, haplotype H58 is a dominant multidrug-resistant lineage that has been reported in endemic regions over the past two decades. We identified fluoroquinolone resistance in cases of *S*. Typhi infection after travel to India. Among them, some strains highly resistant to ciprofloxacin were confirmed to have characteristics of haplotype H58. In Korea, *S*. Typhi haplotype H58 from travelers has not been confirmed before. This study provides information about intercontinental drug-resistant transmission between countries and suggests that travelers need to be careful about personal hygiene.

## Introduction

Typhoid fever, caused by *Salmonella enterica* serovar Typhi (*S*. Typhi), is a systemic disease that causes gastroenteritis, fever, and severe diarrhea. *S*. Typhi is a human-specific pathogen transmitted by the ingestion of contaminated water or food[1]. This disease remains a major burden in developing countries, such as India, Pakistan, and Egypt, and causes 216,000 deaths/year worldwide[2].

Multidrug-resistant (MDR) *S*. Typhi displaying resistance to chloramphenicol, ampicillin, and trimethoprim has been reported in epidemic countries since the 1960s. Resistance to fluoroquinolones and third-generation cephalosporins was recently demonstrated in the 2000s, and more than 90% of South Asian *S*. Typhi strains exhibit decreased susceptibility to ciprofloxacin (CIP)[3]. SNP-based analysis schemes have been developed by stratifying the *S*. Typhi population by haplotype[4]. According to Wong et al., a dominant MDR lineage, *S*. Typhi haplotype H58, initially defined by the SNP *glpA*-C1047T, has become prevalent worldwide over the past two decades. In particular, decreased susceptibility of *S*. Typhi to fluoroquinolones, which is frequently reported in Africa and Asia[5,6], is associated with the H58 sublineage.

Typhoid fever generally occurs in only individuals who have traveled to endemic countries and in immigrants from those regions[7,8]. In Switzerland, quinolone-resistant typhoid infection was reported in a traveler returning from India[9], and in the United Kingdom, MDR *S*. Typhi isolates related to travel to Pakistan have been detected[10]. Typhoid fever is uncommon in the Republic of Korea; approximately 156 cases are reported yearly to the Korea National Surveillance System[11]. In 2017, we first isolated H58 *S*. Typhi during an outbreak involving international travelers returning from India. To investigate the transmission route and characterize the isolates, we analyzed the H58 strain using whole-genome sequencing.

## Materials and methods

### Ethics statement

The Ethics Committee of the First Affiliated Korea Centers for Disease Control and Prevention decided that Institutional Review Board approval was not required. Patient information was collected anonymously and confidential patient information was not included.

### Clinical isolates

Stool and blood samples of patients were processed according to the standard bacterial culture method. Bacterial identification was performed using a VITEK-II automated system (bioMérieux, Marcy l’Etoile, France), and serotyping of *Salmonella* was carried out according to the Kauffmann-White scheme using antisera (BD Biosciences, Frankland Lakes, NJ, USA). The clinical samples were collected anonymously by EnterNet-Korea, a national gastrointestinal infections disease surveillance network.

### Antimicrobial resistance profile

Antimicrobial susceptibilities and minimum inhibitory concentrations (MICs) were determined using the broth microdilution method with 16 antibiotics and customized KRCDC1F Sensititre panels (Trek Diagnostic Systems, OH, USA) in accordance with the guidelines established by the Clinical and Laboratory Standards Institute (CLSI) [12]. The antimicrobial agents tested were Amikacin, Amoxicillin/clavulanic acid 2:1, Ampicillin, Azithromycin, Cefotaxime, Cefoxitin, Ceftazidime, Ceftriaxone, Chloramphenicol, Ciprofloxacin, Gentamicin, Imipenem, Nalidixic Acid, Streptomycin, Tetracycline, Trimethoprim/sulfamethoxazole.

### Whole-genome sequencing

Genomic DNA was isolated with a DNA Blood and Tissue Kit (Qiagen, Hombrechtikon, Switzerland). Whole-genome sequencing was performed using the MiSeq platform (Illumina Inc., San Diego, CA) with a v.2 (500 cycles with 2 x 250-nt reads) kit. The existence of mutations in antibiotic resistance genes and plasmids were determined using Bacterial Analysis Pipeline version 1.0.4. (Illumina BaseSpace Labs, https://basespace.illumina.com/). Other alleles in virulence- and adaptation-related genes were identified using the Geneious Prime 2019.2.1 program. *wgSNP and wgMLST analyses*

Paired-end reads were evaluated with the wgSNP module of BioNumerics v7.6 software (Applied Maths) and mapped to the *S*. Typhi reference genome CT18 (AL513382). Strain-specific SNPs were determined by choosing the default strict filtering option with minimum coverage 30x base quality. Maximum likelihood phylogeny was inferred from the identified SNPs using MEGA-X with 1,000 generalized bootstrap replicates, a time-reversible model, and a gamma distribution with an invariant site. The tree was visualized by iTOL. Next, wgMLST was analyzed using 15,874 loci with the open data set in the wgMLST module. The analysis scheme was developed by Applied Maths, and the default parameters were used. A minimum spanning tree was constructed by advanced cluster analysis.

## Results

### Case description

**Fig 1:**
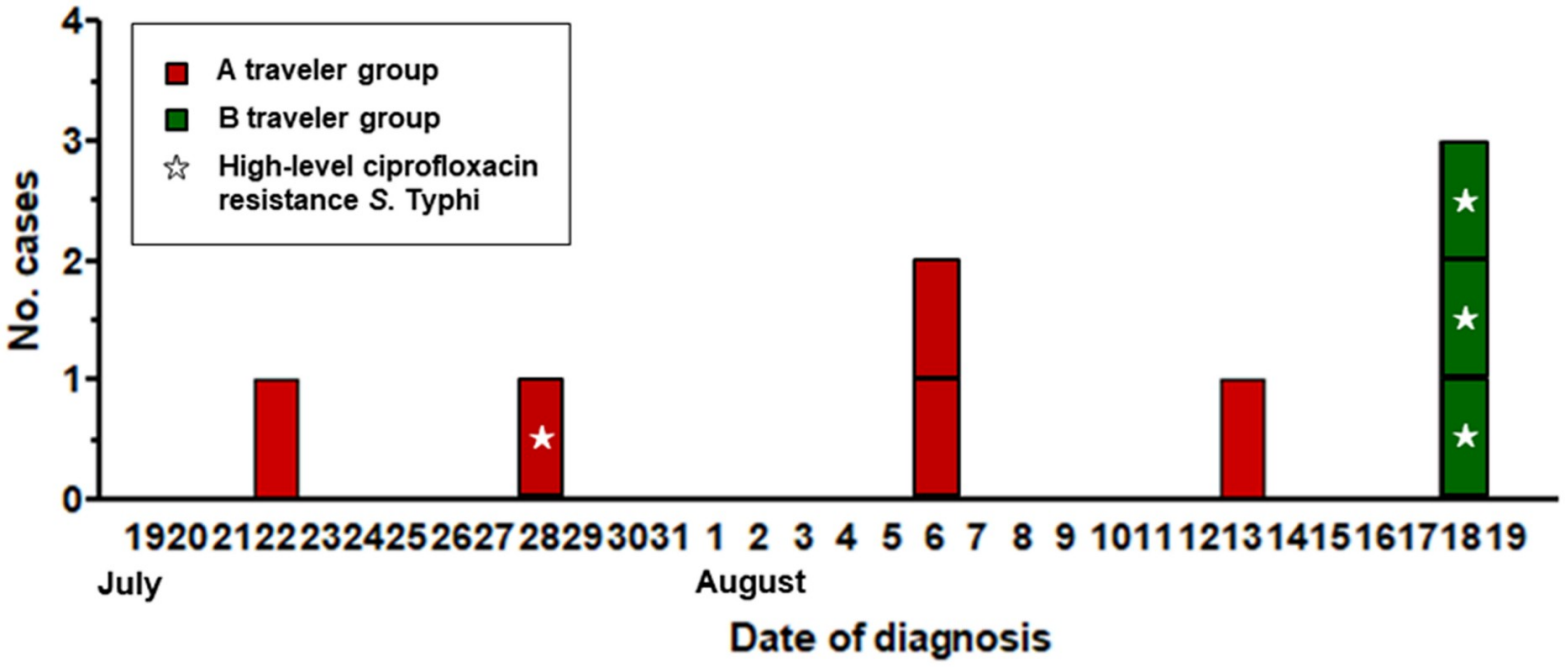
Epidemic curve of the typhoid fever outbreak by date of onset. Traveler group A cases are shown in red, and traveler group B cases are shown in green. Cases of high-level ciprofloxacin-resistant *S*. Typhi are marked with asterisks.

In July and August 2017, eight cases of typhoid fever were identified. Five of the patients had traveled in the northwest region of India (New Delhi, Amritsar, Dharamshala, and Agra) during July 19–27 (group A). The first case occurred on July 22^nd^ and was accompanied by fever, chills, watery diarrhea, headache, and sweating. The onset of the second case occurred on July 28^th^. After returning to Korea, three of the patients showed symptoms such as fever, chills, anorexia and stomachache on the 6^th^ (n = 2) and 13^th^ (n = 1) of August. In August, another group traveled along the same route (group B), and three tourists were reported to have typhoid fever on August 18^th^ (Fig 1). All of the patients subsequently recovered after completing antimicrobial treatment for 8–12 days without complications.

### Antibiotic resistance profiles

*S*. Typhi was isolated from all eight patients, and antimicrobial susceptibility was determined. All strains were resistant to nalidixic acid (NAL; minimum inhibitory concentration (MIC) ≥128 μg/ml) and azithromycin (AZI; MIC ≥8 μg/ml). Only four isolates (1 from group A and 3 from group B) were highly resistant to ciprofloxacin (MIC ≥32 μg/ml) (Table 1). Furthermore, analysis of antimicrobial gene mutations of the CIP-resistant isolates revealed two mutations in the *gyrA* gene (Ser83→Phe (S83F) and Asp87→Asn) and as well as one in the *parC* gene (Ser80→Ile). In addition, analysis of plasmid-mediated quinolone resistance (PMQR) determinants indicated that the CIP-resistant isolates carried the *aac(6’)-Iaa* gene. The non-H58 strain showed a mutation in *gyrA* (S83F), with a PMQR gene (*aac(6’)-Iaa*) located on the plasmid and replicon type IncFIB (pHCM2). Pulsed-field gel electrophoresis (PFGE) results showed identical pattern types for the four CIP-resistant isolates, though the four CIP-susceptible isolates had another PFGE pattern type (supplementary S1 Fig). Notably, neither of the above mentioned PFGE pattern types have been assigned before in PulseNet Korea.

**Table 1:** Antimicrobial resistance profiles and genomic analysis of the outbreak strains.

ID No.	Traveler group	MICs			Antimicrobial Resistance profile	Whole genome analysis					
		CIP	NAL	AZI		Mutants in QRDRs	Screening of PMQRs	Replicon type	MLST	Genotype	Haplotype
KRSAL17-2203	A	0.25	>128	8	NAL, AZI	gyrA(S83F)	aac(6’)-Iaa	IncFIB(pHCM2)	ST2	2.0.2	H50
KRSAL17-2207	A	0.25	>128	8	NAL, AZI	gyrA(S83F)	aac(6’)-Iaa	IncFIB(pHCM2)	ST2	2.0.2	H50
KRSAL17-2205	A	0.25	>128	8	NAL, AZI	gyrA(S83F)	aac(6’)-Iaa	IncFIB(pHCM2)	ST2	2.0.2	H50
KRSAL17-2206	A	0.25	>128	8	NAL, AZI	gyrA(S83F)	aac(6’)-Iaa	IncFIB(pHCM2)	ST2	2.0.2	H50
KRSAL17-2218	B	>32	>128	16	CIP, NAL, AZI	gyrA(S83F,D87N) parC(S80I)	aac(6’)-Iaa		ST1	4.3.1	H58
KRSAL17-2204	A	>32	>128	16	CIP, NAL, AZI	gyrA(S83F,D87N) parC(S80I)	aac(6’)-Iaa		ST1	4.3.1	H58
KRSAL17-2217	B	>32	>128	16	CIP, NAL, AZI	gyrA(S83F,D87N) parC(S80I)	aac(6’)-Iaa		ST1	4.3.1	H58
KRSAL17-2216	B	>32	>128	16	CIP, NAL, AZI	gyrA(S83F,D87N) parC(S80I)	aac(6’)-Iaa		ST1	4.3.1	H58

### Whole-genome sequencing analysis

**Fig 2:**
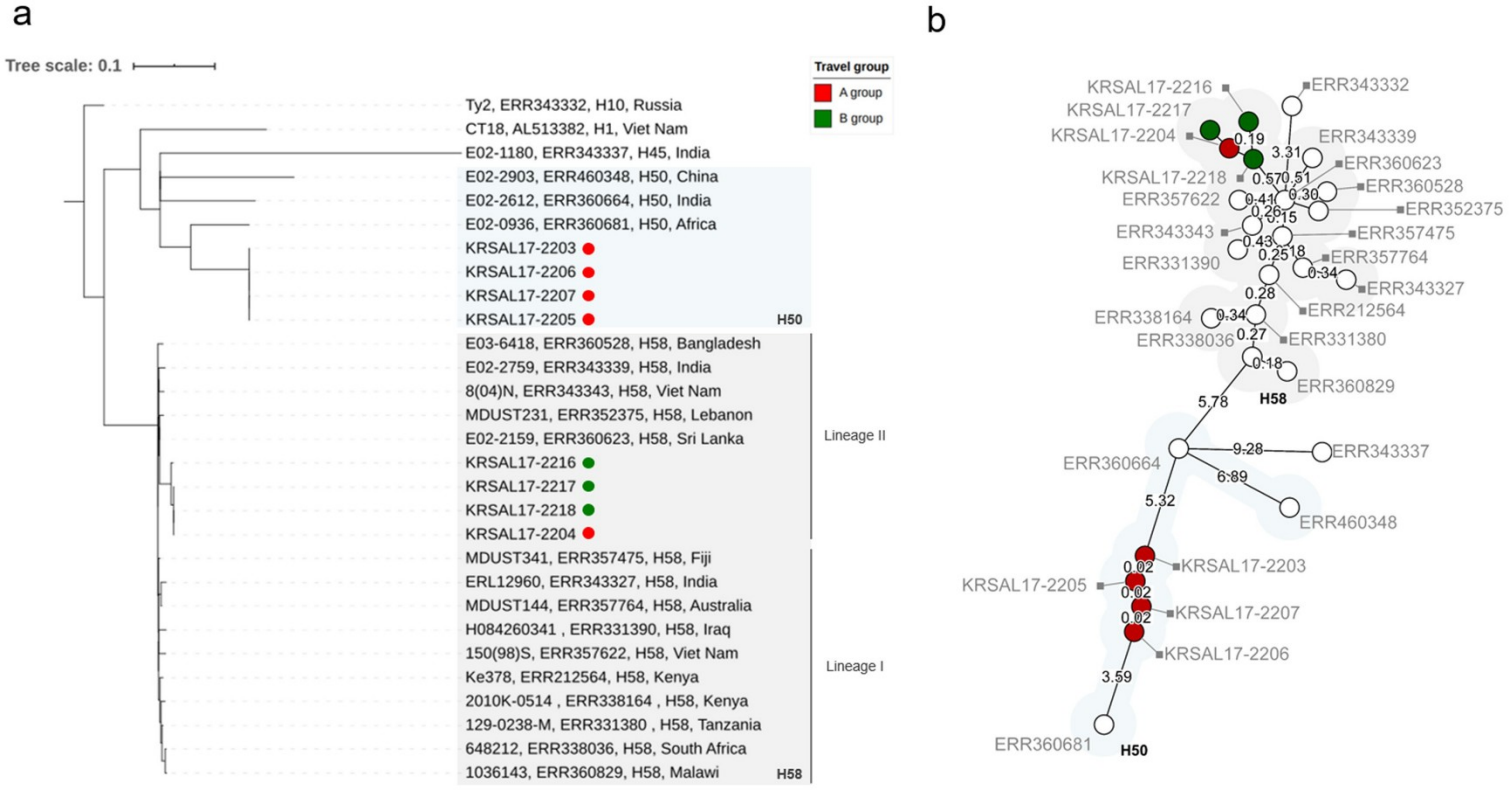
Whole-genome sequencing trees of *S*. Typhi isolates based on SNP and wgMLST analyses. A, Maximum likelihood tree inferred from the 1,577 identified SNPs and visualized by iTOL. Comparative strains from the EBI (European Bioinformatics Institute) database and isolates are marked in the following order: isolate name, Short Read Archive (SRA) accession number, haplotype, and country. The numbers above the nodes represent data from bootstrap replicates. B, Minimum spanning tree based on numerical values (15,874 wgMLST loci) calculated from the core Enterobase, open data set. The branch lengths are represented on a logarithmic scale. Traveler group A cases are shown by red circles, and traveler group B cases are shown by green circles. The cluster of H58 strains is highlighted in light gray, and the H50 strains are highlighted in light blue.

Whole-genome sequencing (WGS) analysis indicated that the high-level CIP-resistant strains belong to haplotype H58 but that the CIP-susceptible strains do not. The CIP-resistant strains displayed the following typical features of H58: SNPs in *glpA* at nucleotide 252 and position 2,348,902 in *S*. Typhi CT18 (C1047T) as well as *SptP* protein Q185* (premature stop codon). Maximum likelihood phylogeny was inferred from 1,577 SNPs (Fig 2A), and SNP-based analysis distinguished the *S*. Typhi isolates by haplotype. The H58 strains formed a tight cluster belonging to sublineage II, with comparative strains from India, Vietnam, Sri Lanka and Lebanon. H58-associated SNPs in membrane and metabolism genes, including *yhdA*, *yajI*, *hyaE*, *tryE*, *rlpB* and *metH*, are present. The non-H58 strains are closely related to haplotype H50. A minimum spanning tree (Fig 2B) based on wgMLST analysis revealed similar results, but it is difficult to distinguish between H58 sublineages.

## Discussion

In Korea, typhoid fever is rare due to improved living standards. Six travel-associated cases (in addition to this outbreak) and 304 *S*. Typhi isolates have been sporadically identified over the past decade[13]. Previous travel cases were associated with China (n = 2), Japan (n = 1), Indonesia (n = 2), and Russia (n = 1). India is a well-known geographical source of H58 with antimicrobial resistance[14]. However, there are no travel cases of typhoid related to epidemic regions in Korea. Therefore, this report describes the initial influx of an *S*. Typhi H58 outbreak following international travel.

Since the 1960s, antimicrobial-resistant *S*. Typhi has been prevalent worldwide. NAL-resistant *S*. Typhi is frequently isolated in India, and high-level CIP-resistant *S*. Typhi was recently isolated in India[15]. In 2007, NAL-resistant *S*. Typhi strains were reported for the first time in Korea, and these isolates did not respond to CIP treatment[16]. These cases suggest the need for alternative treatment such as third-generation cephalosporins or AZI[6]. The isolates showed resistance to NAL and CIP as well as AZI. Moreover, the occurrence of extended-spectrum β-lactamases (ESBLs) harboring a plasmid-mediated Cefotaximase-Munich (CTX-M) 15 gene represented one of the ESBL strains and/or high resistance to quinolones has been reported in other studies[17]. Therefore, an appropriate therapy based on surveillance will be required for future treatment. Indeed, continuous monitoring and data collection will be essential to prevent the occurrence of antimicrobial-resistant *S*. Typhi and treatment failure.

As the dominant *S*. Typhi MDR lineage, haplotype H58 has spread worldwide, particularly in South Asia and Africa[3,4]. In this research, we used WGS analysis to reveal H58 *S*. Typhi infection in international travelers. These H58 strains harbor a *glpA* gene (C1047T) and triple point mutations in quinolone resistance-determining regions (QRDR) such as DNA gyrase (*gyrA* S83F D87N) and topoisomerase IV (*parC* S80I). Usually, CIP-resistant, intermediate *S*. Typhi isolates harbor a single mutation in *gyrA* and CIP highly resistant isolates have triple point mutations similar to our strain[18]. Most H58 lineages are related to reduced global susceptibility to fluoroquinolones and the presence of IncHI1[4]. However, our H58 strains lack the MDR plasmid from the IncHI1 type. Additionally, we detected a point mutation in *SptP*, resulting in a premature stop codon at position 185. There is one genomic signature in H58[4]. The degradation of *SptP*, a type III secretion system (T3SS) effector within *Salmonella* pathogenicity island-1(SPI-1), resulted in invasion, virulence and intestinal colonization defects[19]. The genetic mutations are related to a major metabolism mechanism, fluoroquinolone resistance. We examined the mutations of efflux pump-related genes. Mutations in *AcrA*-T270S, *AcrB*-T964M, and *TolC*-T293A, D457E were confirmed, but are still unknown. Further studies will be need to define whether these mutations cause critical changes and affect antimicrobial resistance.

Fig 2. shows the results of comparative analysis by wgSNP and wgMLST. Both phylogenetic trees look similar. The maximum likelihood tree was generated from wgSNP analysis (Fig 2A) by comparing with the CT18 reference strain and is mainly used to distinguish the haplotype. The minimum spanning trees were calculated using the wgMLST scheme from Core EnteroBase. Based on the PulseNet network, we tried to accumulate an allele database from wgMLST for global standardization. In our H58 strain, the difference of 0.19 of the log value of KRSAL17-2216 is approximately 4 SNPs (Fig 2B). The wgMLST results seem similar to the wgSNP analysis, but also are able precisely to identify and distinguish the genotype.

Additionally, we confirmed newly reported *S*. Typhi isolates related to travelers [20]. The SRR5584965 (India, 2017) and SRR5585105 (Pakistan, 2017) strains are closely related to our H58 strain. They have almost similar genome sequences (difference of ~10 SNPs). We have been assured that our H58 strains have already been epidemic in India and Pakistan in 2017. Strangely, KRSAL17-2204 of group A has similar association to persons of Group B with high level ciprofloxacin resistance as H58. As reported in the epidemiological investigation, it was confirmed that the two groups traveled the same route on different days, but details such as whether there had been contact between the tour groups or the tour groups visited the same place or ate the same food were not confirmed. Around the same time, there was a case of typhoid in India infected by contaminated water[18]. Additionally, these cases are common in India. By our guess, the travelers were exposed to the same poor sanitation or contaminated water environment.

Non-H58 strains, like H50 strains, have less antibiotic resistance than H58 strains and studies focused on them are lacking[3,21]. Our studies determined that isolated H50 strains have resistance to NAL and AZI. The results suggest that the prevalence of the distribution of antibiotic resistant strains increase over time. Moreover, the fluoroquinolone-resistant H58 with resistance to other antimicrobials is still circulating in epidemic regions. Our report emphasizes that continuous management regardless haplotypes through international surveillance networks such as the PulseNet network, education of traveler personal hygiene and adequate pathogen precaution will need. Thus, continuous surveillance is needed to monitor intercontinental transmission of antimicrobial-resistant strains, and support the global management of typhoid fever.

## Supporting information

S1 Fig The XbaI-PFGE patterns of the outbreak strains. The dendrogram was constructed using the Dice coefficient and UPGMA clustering with 1.5% optimization and 1.5% position tolerance. PFGE pattern numbers (SPPX01.215–216) newly assigned by PulseNet Korea.
(TIF)

## References

[1] Crump JA, Mintz ED. Global Trends in Typhoid and Paratyphoid Fever. Clin Infect Dis. 2010;50(2):241–6. 10.1086/649541 20014951 PMC2798017

[2] Mogasale V, Maskery B, Ochiai RL, Lee JS, Mogasale V V., Ramani E, et al. Burden of typhoid fever in low-income and middle-income countries: A systematic, literature-based update with risk-factor adjustment. Lancet Glob Heal [Internet]. 2014;2(10):e570–80. Available from: 10.1016/S2214-109X(14)70301-8 25304633

[3] Dyson ZA, Klemm EJ, Palmer S, Dougan G. Antibiotic Resistance and Typhoid. Clin Infect Dis. 2019;68(2):S165–70. 10.1093/cid/ciy1111 30845331 PMC6405283

[4] Wong VK, Baker S, Pickard DJ, Parkhill J, Page AJ, Feasey NA, et al. Phylogeographical analysis of the dominant multidrug-resistant H58 clade of Salmonella Typhi identifies inter-and intracontinental transmission events. Nat Genet. 2015;47(6):632–9. 10.1038/ng.3281 25961941 PMC4921243

[5] Park SE, Pham DT, Boinett C, Wong VK, Pak GD, Panzner U, et al. The phylogeography and incidence of multi-drug resistant typhoid fever in sub-Saharan Africa. Nat Commun. 2018;9(1). 10.1038/s41467-018-07370-z 30504848 PMC6269545

[6] Thanh DP, Karkey A, Dongol S, Thi NH, Thompson CN, Rabaa MA, et al. A novel ciprofloxacin-resistant subclade of h58. Salmonella typhi is associated with fluoroquinolone treatment failure. Elife. 2016;5(MARCH2016):1–13. 10.7554/eLife.14003 PMC4805543 26974227

[7] Steinberg EB, Bishop R, Haber P, Dempsey AF, Hoekstra RM, Nelson JM, et al. Typhoid fever in travelers: Who should be targeted for prevention? Clin Infect Dis. 2004;39(2):186–91. 10.1086/421945 15307027

[8] O’Brien DP, Leder K, Matchett E, Brown G V., Torresi J. Illness in returned travelers and immigrants/refugees: The 6-year experience of two Australian infectious diseases units. J Travel Med. 2006;13(3):145–52. 10.1111/j.1708-8305.2006.00033.x 16706945

[9] Nüesch-Inderbinen M, Abgottspon H, Sägesser G, Cernela N, Stephan R. Antimicrobial susceptibility of travel-related Salmonella enterica serovar Typhi isolates detected in Switzerland (2002–2013) and molecular characterization of quinolone resistant isolates. BMC Infect Dis. 2015;15(1):1–5. 25963025 10.1186/s12879-015-0948-2 PMC4435775

[10] Chatham-Stephens K, Medalla F, Hughes M, Appiah GD, Aubert RD, Caidi H, et al. Emergence of Extensively Drug-Resistant Salmonella Typhi Infections Among Travelers to or from Pakistan—United States, 2016–2018. MMWR Morb Mortal Wkly Rep. 2019;68(1):11–3. 10.15585/mmwr.mm6801a3 30629573 PMC6342547

[11] Korea Centers for Disease Control and Prevention (KCDC). Incidence of Legal infectious disease in national monitoring index [Internet]. http://www.index.go.kr/potal/main/EachDtlPageDetail.do?idx_cd=1442

[12] Clinical and Laboratory Standards Institute (CLSI) Performance standards for antimicrobial susceptibility testing. M100 28th edition. Wayne, PA. 2018.

[13] Korea Centers for Disease Control and Prevention (KCDC). Korea infectious disease portal [Internet]. http://www.cdc.go.kr/npt/biz/npp/ist/bass/bassDissStatsMain.do

[14] Wirth T. Massive lineage replacements and cryptic outbreaks of Salmonella Typhi in eastern and southern Africa. Nat Genet. 2015;47(6):565–7. 10.1038/ng.3318 26018894

[15] Renuka K, Sood S, Das BK, Kapil A. High-level ciprofloxacin resistance in Salmonella enterica serotype Typhi in India. Vol. 54, Journal of Medical Microbiology. 2005. p. 999–1000. 10.1099/jmm.0.45966-0 16157558

[16] Kim D-B, Kim S-H, Oh S-J, Kim D-K, Choi S-M, Kim M-S, et al. Three cases of ciprofloxacin treatment failure in imported typhoid fever. Korean J Med. 2009;77(3):377–81.

[17] Klemm EJ, Shakoor S, Page AJ, Qamar FN, Judge K, Saeed DK, et al. Emergence of an extensively drug-resistant Salmonella enterica serovar typhi clone harboring a promiscuous plasmid encoding resistance to fluoroquinolones and third-generation cephalosporins. MBio. 2018;9(1):1–10. 10.1128/mBio.00105-18 29463654 PMC5821095

[18] India: Two dozen typhoid cases reported in Krishna district, Vijayawada [Internet]. Promed. 2017. https://promedmail.org/promed-posts/, Archive Number: 20170808.5235812

[19] Johnson R, Byrne A, Berger CN, Klemm E, Crepin VF, Dougan G, et al. The type III secretion system effector SptP of Salmonella enterica serovar Typhi. J Bacteriol. 2017;199(4):1–18. 10.1128/JB.00647-16 27920299 PMC5287405

[20] Ingle DJ, Nair S, Hartman H, Ashton PM, Dyson ZA, Day M, et al. Informal genomic surveillance of regional distribution of Salmonella Typhi genotypes and antimicrobial resistance via returning travellers. PLoS Negl Trop Dis [Internet]. 2019;13(9):1–20. Available from: 10.1371/journal.pntd.0007620 31513580 PMC6741848

[21] Holt KE, Dolecek C, Chau TT, Duy PT, la TTP, van Minh Hoang N, et al. Temporal fluctuation of multidrug resistant Salmonella typhi haplotypes in the mekong river delta region of Vietnam. PLoS Negl Trop Dis. 2011;5(1):1–10. 10.1371/journal.pntd.0000929 21245916 PMC3014949

