# Supplementary figures and images for: Emerging high-level ciprofloxacin-resistant Salmonella enterica serovar typhi haplotype H58 in travelers returning to the Republic of Korea from India
Source: PLoS Negl Trop Dis. 2021 Mar 2;15(3):e0009170. doi: 10.1371/journal.pntd.0009170 (PMC7987170; doi:10.1371/journal.pntd.0009170)

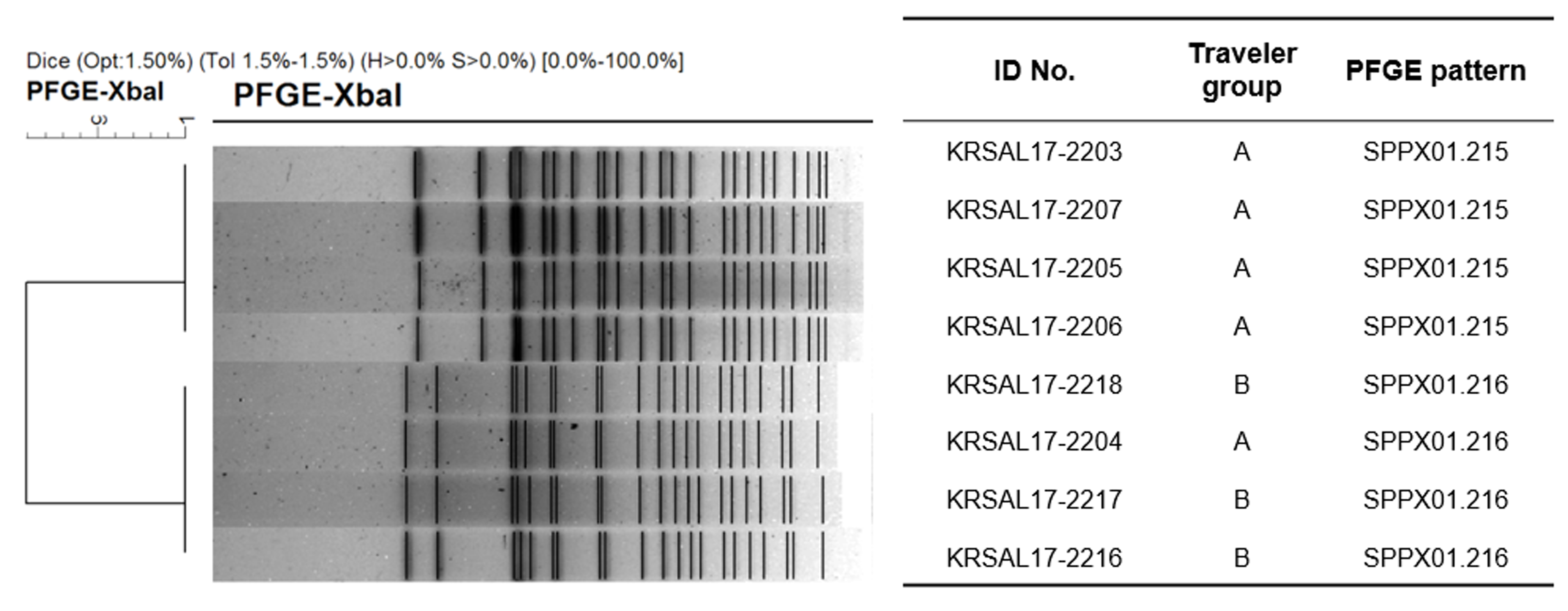

Supplement: S1 Fig — The dendrogram was constructed using the Dice coefficient and UPGMA clustering with 1.5% optimization and 1.5% position tolerance. PFGE pattern numbers (SPPX01.215–216) newly assigned by PulseNet Korea. (TIF) [file pntd.0009170.s001.tif]
